# Antibacterial effect of cerium oxide nanoparticle against *Pseudomonas aeruginosa*

**DOI:** 10.1186/s12896-021-00727-1

**Published:** 2021-12-07

**Authors:** Khosro Zamani, Noushin Allah-Bakhshi, Faezeh Akhavan, Mahdieh Yousefi, Rezvan Golmoradi, Moazzameh Ramezani, Horacio Bach, Shabnam Razavi, Gholam-Reza Irajian, Mahyar Gerami, Ali Pakdin-Parizi, Majid Tafrihi, Fatemeh Ramezani

**Affiliations:** 1 1. Microbial Biotechnology Research Center, Iran University of Medical Sciences, Tehran, Iran. 2. Department of Microbiology, School of Medicine, Iran University of Medical Sciences, Tehran, Iran; 2 Department of Biology, Sana Institute of Higher education, Sari, Iran; 3 Department of Biology, Urmia University, Urmia, Iran; 4 Division of Infectious Diseases, Department of Medicine, University of British Columbia, Vancouver, BC Canada; 5 Sari Agricultural Sciences and Natural Resources University, Sari, Iran; 6 Department of Molecular and Cell Biology, Faculty of Basic Sciences, University of Mazandaran, Babolsar, Mazandaran Iran; 7 Physiology Research Center, Iran University of Medical Sciences, Tehran, Iran

**Keywords:** Cerium oxide nanoparticles, Nanofiber, Antibiotic resistance, *Pseudomonas aeruginosa*, Gene expression, Cytotoxicity, Clinical isolate

## Abstract

**Background:**

Antibiotics have been widely used for the treatment of bacterial infections for decades. However, the rapid emergence of antibiotic-resistant bacteria has created many problems with a heavy burden for the medical community. Therefore, the use of nanoparticles as an alternative for antibacterial activity has been explored. In this context, metal nanoparticles have demonstrated broad-spectrum antimicrobial activity. This study investigated the antimicrobial activity of naked cerium oxide nanoparticles dispersed in aqueous solution (CNPs) and surface-stabilized using *Pseudomonas aeruginosa* as a bacterial model.

**Methods:**

Gelatin-polycaprolactone nanofibers containing CNPs (Scaffold@CNPs) were synthesized, and their effect on *P. aeruginosa* was investigated. The minimum inhibitory and bactericidal concentrations of the nanoparticls were determined in an ATCC reference strain and a clinical isolate strain. To determine whether the exposure to the nanocomposites might change the expression of antibiotic resistance, the expression of the genes *shv*, *kpc*, and *imp* was also investigated. Moreover, the cytotoxicity of the CNPs was assessed on fibroblast using flow cytometry.

**Results:**

Minimum bactericidal concentrations for the ATCC and the clinical isolate of 50 µg/mL and 200 µg/mL were measured, respectively, when the CNPs were used. In the case of the Scaffold@CNPs, the bactericidal effect was 50 µg/mL and 100 µg/mL for the ATCC and clinical isolate, respectively. Interestingly, the exposure to the Scaffold@CNPs significantly decreased the expression of the genes *shv*, *kpc*, and *imp*.

**Conclusions:**

A concentration of CNPs and scaffold@CNPs higher than 50 μg/mL can be used to inhibit the growth of *P. aeruginosa*. The fact that the scaffold@CNPs significantly reduced the expression of resistance genes, it has the potential to be used for medical applications such as wound dressings.

## Background

Nosocomial infection is one of the most important medical problems in developed and developing countries [1, 2]. Antibiotics have been widely used for the treatment of bacterial infections for decades. However, the rapid emergence of antibiotic-resistant bacteria has created many problems and burdens for the medical community [3, 4]. Each year, approximately 88,000 deaths from hospital-acquired infections are reported in the United States [5]. Treatment of tuberculosis and pneumonia has become more difficult because of the appearance of resistant strains, with the consequences of more extended hospitalizations [6]. *Pseudomonas aeruginosa* is one of the most common causes of hospital-acquired infections with severe or fatal outcomes, especially in immunocompromised hosts. This opportunistic bacterium infects soft tissues and injured skin, including burn wounds [7, 8]. Complications of *P. aeruginosa* can lead to meningitis, pneumonia, and other deadly diseases [9, 10]. Extensive use of antibiotics in recent years has made this bacterium resistant to broad-spectrum antibiotics [11, 12].

A promising alternative to combat bacterial resistance comes from metal nanoparticles (NPs) [13, 14]. NPs have high chemical and biological activity due to different factors, mainly their small size and their high surface-to-volume ratio [15–17]. As a result, they have been widely used in biology and medicine [18–22].

Metal NPs target different bacterial macromolecules and disrupt the normal function of the cell membrane, including selective permeability and cellular respiration [23–25]. In addition, possible interactions of positive-charged NPs with the negative charge macromolecules on the surface of microorganisms can drive an electrostatic force for absorption of the NPs on the cell surface with a detrimental effect on the survival of the cell [24, 26]. Furthermore, NPs can control and stop the cell cycle by interfering with enzymes involved in bacterial proliferation and through gene-toxicity and the potential for the generation of gene mutation(s) [27, 28].

Many studies have shown that cerium oxide nanoparticles (CNPs) exhibit excellent antimicrobial activity [4, 29, 30]. The antibacterial effect of CNPs on *Staphylococcus aureus* was demonstrated in various studies [31–33], including a potent antibacterial effect [34–37]. Moreover, several studies evaluated and verified the *P. aeruginosa* sensitivity to CNPs by agar diffusion and microdilution tests [32, 38, 39]. Although the antibacterial activity of CNPs against different strains of bacteria has been reported, the expression of resistance genes related to the antibacterial effect of CNPs has not been investigated so far.

The use of suitable wound dressing materials, especially those derived from biopolymers, could reduce the incidence of infection and accelerate the healing process. In particular, biocompatible and highly degradable nanofiber dressings that mimic the extracellular matrix structure can provide high surface area for a focal delivery of antibacterial agents to control infection [38–41].

In this study, we investigated the antibacterial properties of naked and nanofiber-immobilized (scaffold) CNPs using *P. aeruginosa* as a bacterial model. We also analyzed the effect of the CNPs on the expression of the β-lactamase *shv*, the carbapenemase *kpc*, and the metallo-β-lactamase *imp* genes. To demonstrate the biocompatibility of the CNPs, a cytotoxic assay was conducted using a model of skin fibroblast cells.

## Material and methods

### Bacterial strain

*P. aeruginosa* (ATCC 27853) was obtained from the microbial collection of the microbiology laboratory of Iran University of medical sciences. A clinical isolate of the same strain was obtained from an infected burn of a patient at the Ali-Asghar hospital in Tehran, Iran.

### Naked and scaffold- CNPs synthesis

CNP powder was purchased from Sigma-Aldrich (Cat. # 796077). Nanofibers were fabricated by mixing 80 mL of chloroform with 4 g of polycaprolactone under a magnetic stirrer for 4 h. Then, a gelatin/acetic acid solution (1.6 g of gelatin and 20 mL of 80% acetic acid) was added to the mixture. Nanofibers were produced by an electrospinning device (Fanavaran Nano-Meghyas, IRAN) at 60% power for 1 h with rotation at 30 °C using a voltage of 20 kV and a speed of 10 µL/min using a 10 cm nozzle. An aluminum collector and a rotating core were used at 450 × *g* to obtain random-axis nanofibers exposed to different concentrations of the following CNPs solutions: P: 200 µg/mL, P/2: 100 µg/mL, P/4: 50 µg/mL, P/8: 25.5 µg/mL, P/16: 12.25 µg/mL, P/32: 6.125 µg/mL overnight. After coating the samples with gold, the final Scaffold@CNPs structure was imaged using a scanning electron microscope (SEM, DSM-960A Zeiss, Carl Zeiss, Germany). Energy Dispersive X-ray (EDX system Kevex) spectroscopy was performed to identify the elements in the nanofiber.

### In vitro release of CNPs

To investigate the release of CNPs from the scaffold, the nanocomposite was immersed in PBS at 37 °C for 9 days. The optical density of the samples was measured at 300–350 nm [42–45] using a UV–Vis spectrophotometer (Thermo Fisher Scientific, Waltham, Massachusetts, USA) on days 1, 3, 5, 7, and 9. Experiments were performed in triplicate.

### Antibacterial activity

#### Minimum inhibitory concentration (MIC)

A microdilution test was used to determine the MICs. The experiment was performed in sterile 96-well plates containing 100 µL of Muller-Hinton broth (M-H). CNPs concentrations of 100, 50, 25, 12.5, 6.25, 3.12, 1.56, 0.78, 0.39, 0.195 µg/mL were tested. M-H broth and untreated bacteria were used as negative and positive controls, respectively. The Scaffold@CNPs P, P/2, P/4, P/8, P/16, and P/32 were tested in a second microplate.

A suspension of bacteria corresponding to 0.5 McFarland unit was prepared, and after a dilution of 20X, 10 µL was added to each well (approximately 5 × 10^4^ CFU/mL). Plates were incubated at 37 °C for 24 h. The results were evaluated based on the lack of growth or significant growth of bacteria in the wells. The lowest concentration of NPs that inhibited the growth of the microorganism was recorded as the MIC.

#### Minimum bactericidal concentration (MBC)

The final CNP concentration that showed no bacterial growth (no turbidity observed in the MIC test) was cultured on M-H agar and incubated at 37 °C for 24 h after serial dilution. The next day, the colonies were counted.

#### Investigation of resistance genes using real-time PCR

The resistant clinical isolate was grown on M-H broth containing 50 µg/mL or 200 µg/mL of CNPs or Scaffold@CNPs, respectively. Kanamycin (4 µg/mL) was added for 24 h. Total RNA was extracted from bacteria using the RNX^+^ extraction kit (Cinagen Bioscience, Tehran, Iran) and following the manufacturer’s instructions. DNAse was used to digest DNA remnants. The RNA concentration was measured using a Nano-Drop instrument (Thermo Scientific). The oligonucleotide sequences used in this study are detailed in Table 1. The 16S ribosomal RNA from *P. aeruginosa* was used as an internal control. The qPCR reaction (20 µL) used the Maxima SYBR green kit (Thermo Scientific) and according to the manufacturer’s instructions. A thermocycler (ABI, USA)was operated using a program consisting of 1 × cycle of 95 °C for 5 min, followed by 40 × cycles of 95 °C for 30 s and 60 °C for 40 s.

**Table 1:** Oligonucleotide sequences used in the gene expression analysis

Gene	Oligonucleotide	Sequence	Tm (°C)	GC (%)
*shv*	Forward	TTCTATCATGCCTACGCGGC	60. 32	55. 00
	Reverse	ATCTCCCTGTTAGCCACCCT	59. 96	55. 00
*imp*	Forward	AAGAAGTTAACGGGTGGGGC	60. 25	55. 00
	Reverse	CACGCTCCACAAACCAAGTG	59. 97	55. 00
*kpc*	Forward	TGTGTACGCGATGGATACCG	59. 97	55. 00
	Reverse	TTTTGCCGTAACGGATGGGT	60. 25	50. 00
*16S*	Forward	CCACGCCACTGATCTTCCAT	60.11	55.00
	Reverse	CTGGACCATGATCGAGAGCC	59.97	60.0

### Cytotoxicity

Scaffold@CNPs were exposed to human foreskin fibroblast HU2 cells obtained from the Iranian Biological Resource Center (Tehran, Iran) for 1, 3, and 7 days at 37 °C in an incubator supplemented with 5% CO_2_. Dulbecco's Modified Eagle Medium (DMEM) medium, supplemented with 10% fetal bovine serum (FBS) and 1% Penicillin/Streptomycin.

Apoptotic cells were identified using the Annexin V-propidium iodide (PI) staining kit (640914, Biolegend). 6-well plates were seeded with 3 × 10^5^ cells and incubated for 24 h at 37 °C. The next day, the medium was changed and replaced with 4 mL of culture medium containing 150 µL of P, P/2, P/4, P/8, and P/16. A similar plate was used, but the CNPs replaced the Scaffold@CNPs. The plates remained in the incubator for 24 h. The next day, 400 µL of trypsin was added, and once the cells detached, 400 µL of fetal bovin serum (FBS)-containing medium were added to each well. The content of each well was centrifuged at 20,000 rpm for 5 min, and the supernatant was disposed. Then, 100 µL of PBS was added. Annexin-V solution was added and incubated for 10 min in a dark place. The samples were centrifuged, and cells were rinsed with PBS. Then, 1.5 µL of PI was added. The samples were analyzed with the flow cytometer.

### Statistical analysis

Statistical analysis was performed using SPSS and a one-way ANOVA test. Excel was used to draw the graphs. Values are reported as the mean ± SD of three independent experiments.

## Results

### Characterization of the nanoparticles

**Fig. 1:**
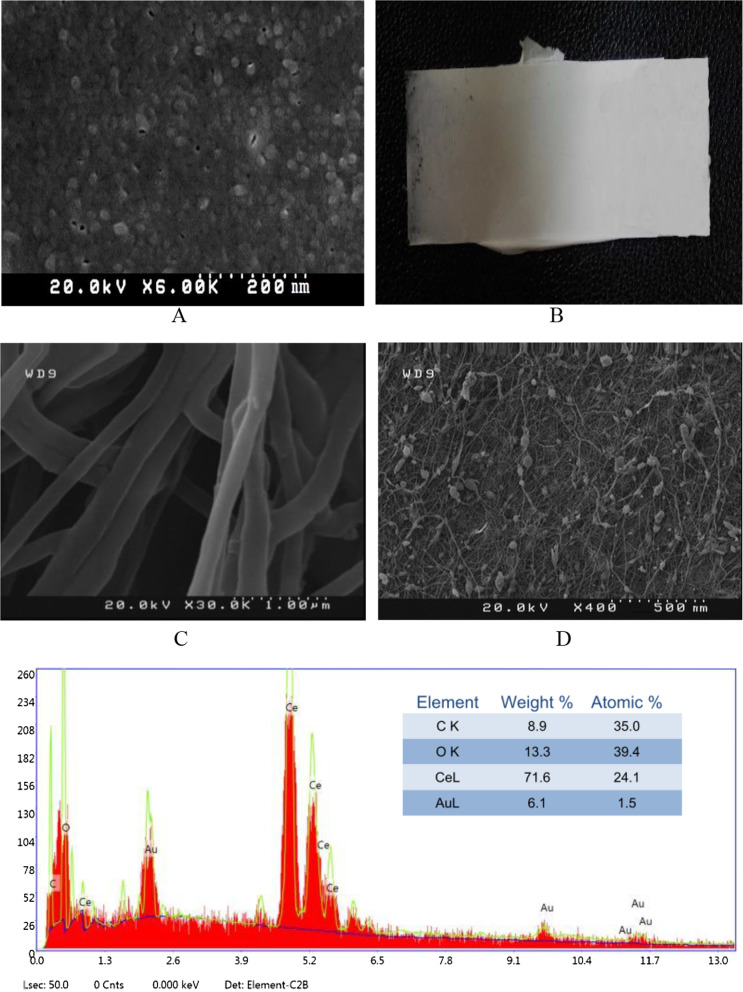
Characterization of CNPs and Scaffold@CNPs. **A** SEM image of CNPs, **B** Appearance of nanofibers containing 5% PCL, **C** SEM image of nanofiber without CNPs, **D** SEM image of Scaffold@CNPs, and **E** EDX analysis

A zeta potential of + 18 mV was measured. In addition, SEM images confirmed that the CNPs were spherical with a size range ≤ 20 nm (Fig. 1A). The formation of the Scaffold@CNPs and the diameter and scale of the fibers are shown in Fig. 1B and C. Moreover, the presence of the CNPs on the surface of the fibers was confirmed by SEM imaging (Fig. 1D). Analysis of the peaks in the spectrum obtained from EDX confirmed the presence of cerium in the NPs (Fig. 1E).

**Fig. 2:**
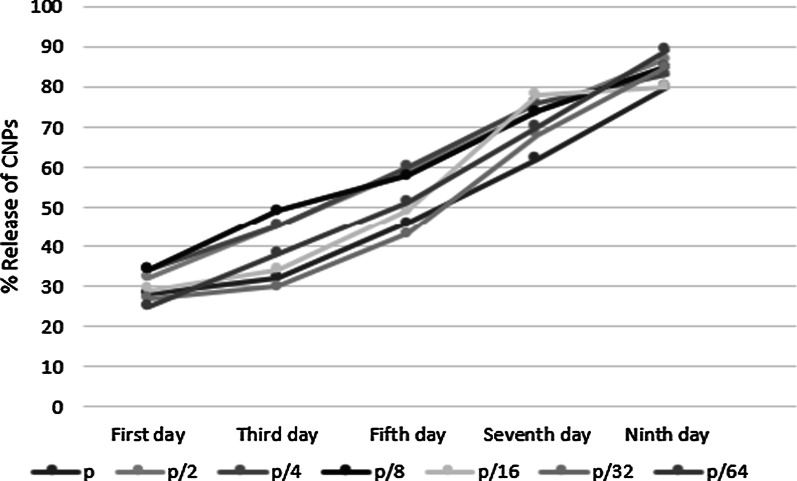
Release of CNPs from Scaffold@CNPs containing P: 200 µg/mL, P/2: 100 µg/mL, P/4: 50 µg/mL, P/8: 25.5 µg/mL, P/16: 12.25 µg/mL, and P/32: 6.125 µg/mL over a period of 9 days. The samples were measured by a UV–Vis spectrophotometer using a wavelength between 300 and 350 nm

Scaffold@CNPs containing P, P/2, P/4, P/8, P/16, and P/32 were used to study the CNPs release from the nanofiber. A release ranging between 25 and 35% was measured on day 1, with a concomitant increase to 80–90% measured on day 9 (Fig. 2). In summary, according to the CNP release pattern from the Scaffold@CNPs, it is expected that 25–35% of the total amount of CNP will be released on the first day.

### Antimicrobial effect of CNPs and Scaffold@CNPs

The CNPs showed MICs of 12.5 µg/mL for both the ATCC and the clinical isolate, but an MBC of 200 µg/mL was necessary to kill the clinical isolate (Table 2). For the Scaffold@CNPs, MICs of 6.25 µg/mL µg and 12/mL could inhibit the growth of the ATCC and clinical isolate, respectively. Moreover, MBCs of 50 µg/mL and 100 µg/mL were necessary to kill the bacterial cells (Table 2).

**Table 2:** MICs and MBCs of CNPs and Scaffold@CNPs against *P. aeruginosa* strains expressed in µg/mL

Strain	CNPs		Scaffold@CNPs	
	MIC	MBC	MIC	MBC
ATCC strain	12.5	50	6.25	50
Clinical isolate	12.5	200	12.5	100

### Evaluation of resistance genes expression in *P. aeruginosa* to CNPs

**Fig. 3:**
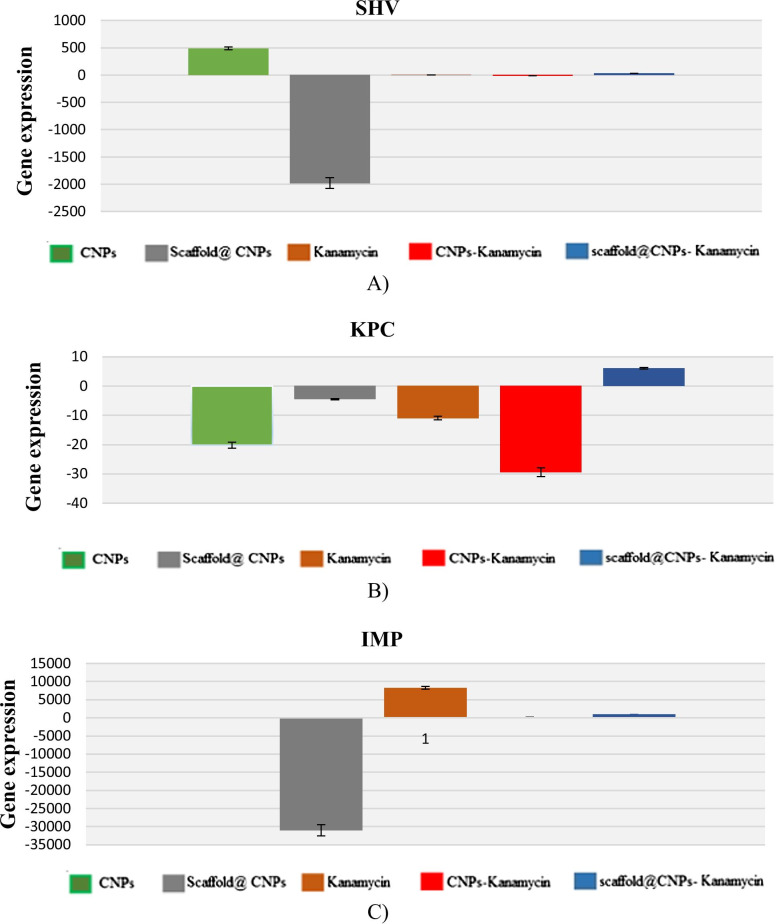
Expression of genes conferring antibiotic resistance to *P. aeruginosa*. Bacterial cells were harvested under different treatments, and the total RNA was converted into cDNA according to Materials and Methods. **A** *shv*, **B** *kpc*, and **C** *imp* genes. Shown are the mean ± SD of three independent samples

The levels of the three genes *shv*, *kpc*, and *imp*, which are related to antibiotic resistance in *P. aeruginosa*, were evaluated after exposure of the bacterial cells to different combinations of the nanocomposites and the antibiotic kanamycin. The combinations used were: (1) CNPs, (2) Scaffold@CNPs, (3) kanamycin, CNPs + kanamycin, and (4) Scaffold@CNPs + kanamycin. The gene *shv* was down regulated after treating the cells with Scaffold@CNPs, but an up-regulation was measured when the CNPs alone were used (Fig. 3A). No changes were observed in the other treatments. In the gene *kcp*, most treatments showed a downregulation of the gene except for the Scaffold@CNPs + kanamycin group (Fig. 3B). Lastly, a significant downregulation was measured in the Scaffold@CNPs, but not in the other treatments, except under the presence of kanamycin (Fig. 3C).

### Cytotoxicity of CNPs and Scaffold@CNPs

**Fig. 4:**
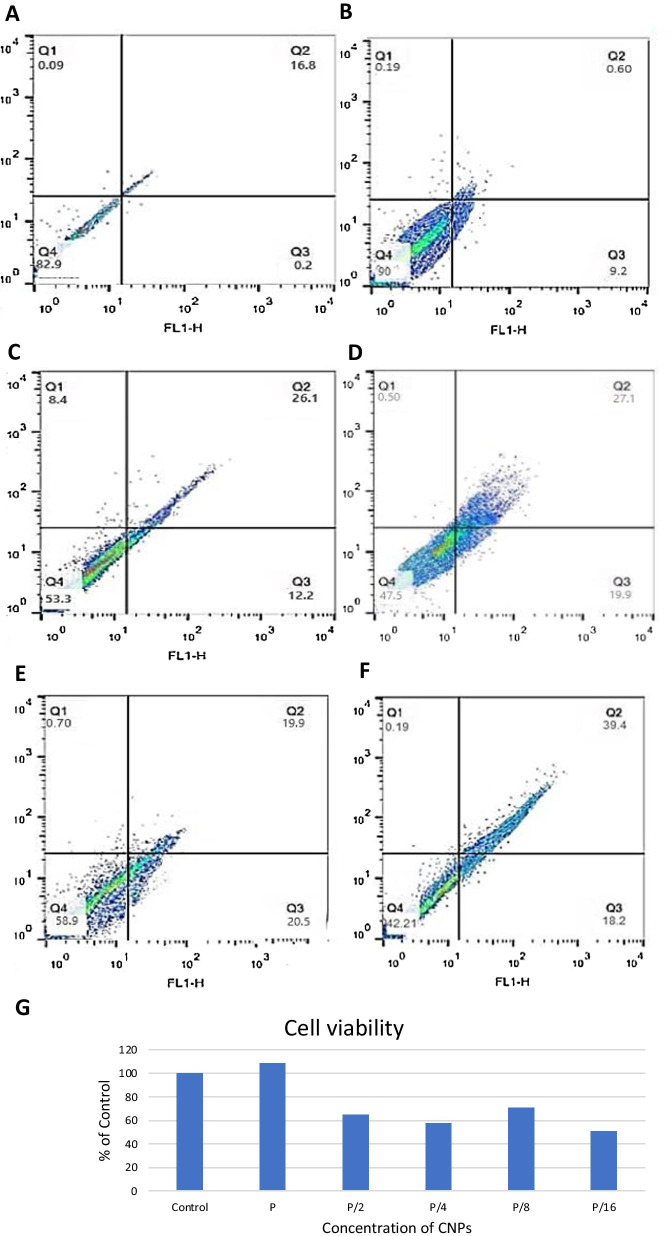
Cytotoxicity analysis of HU2 cell line exposed to the CNPs. Analysis was performed by flow cytometry and according to Materials and Methods. **A** Control, **B** P, **C** P/2, **D** P/4, **E** P/8, **F** P/16, and **G** Survival rate of the cells normalized to the control. Q1 = Necrotic cells, Q2 = Late apoptotic or necrotic apoptotic cells, Q3 = Apoptotic cells, and Q4 = Untreated cells

Cytotoxicity of human fibroblast cells (HU2 cell line) after exposure to CNPs is shown in Fig. 4. Results of the fluorescence of all the quarters are summarized in Fig. 4G.

**Fig. 5:**
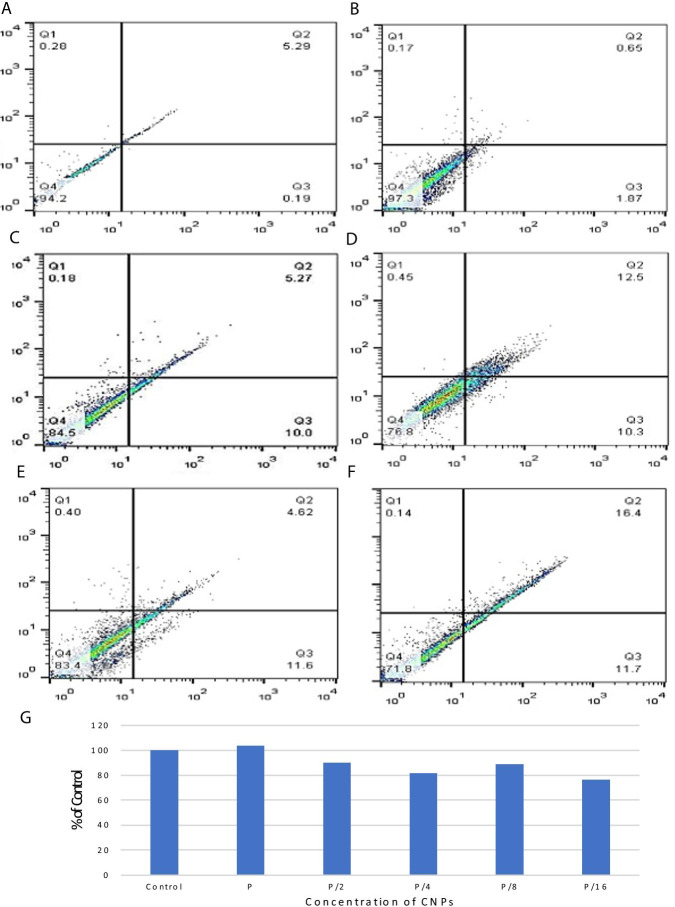
Cytotoxicity analysis of HU2 cell line exposed to the Scaffold@CNPs. Analysis was performed by flow cytometry and according to Materials and Methods. **A** Control, **B** P, **C** P/2, **D** P/4, **E** P/8, **F** P/16, and **G** Survival rate of the cells normalized to the control. Q1 = Necrotic cells, Q2 = Late apoptotic or necrotic apoptotic cells, Q3 = Apoptotic cells, and Q4 = Untreated cells

The cytotoxicity results fibroblast cells exposed to Scaffold@CNPs can be seen in Fig. 5. Figure 5a shows 94% of control untreated cells remained unstained l. Figure 5b represents in presence of Scaffold@CNPs containing 200 μg/ml of CNPs, 97% of the cells survive.

Figure 5c shows that at P/2 concentration, the number of living cells reduced to 84%, and 5% in the death stage and 10% in the early stage of death. By reducing the concentration of nanoparticles in Scaffold@CNPs, Fig. 5d shows that in the P/4 concentration the number of living cells decreased to 76% and 12% of cells were in the death stage and 10% in the early stage of death. The effect of Scaffold@CNPs with P/8 and P/16 concentrations is presented in Fig. 5e and f. The number of living cells in these two concentrations were 83% and 71%, respectively.

## Discussion

This study aimed to investigate the antibacterial properties of naked and fixed CNP-containing nanofibers as a potential used to treat *P. aeruginosa*.

Results showed that CNPs in solution at concentrations ranging between to 200 µg/mL had an inhibitory effect on the ATCC and the clinical isolate of *P. aeruginosa*. These results are consistent with the findings of a previous study, which showed MICs of 20 ± 5 μg/mL [4]. Moreover, the inhibitory effect of CNPs was also demonstrated on Gram-negative bacteria such as *E.coli* [46, 47] and *Klebsiella pneumoniae* [48].

The clinical isolate used in our study was isolated from burn patients receiving antibiotics but suffering from an antibiotic-induced infection. Our study aligned with the fact that clinical isolates are more resistant to antibiotics than the ATCC strains. Thus, clinical isolates isolated from hospitals are more resistant to antibiotics, likely because of the acquisition of plasmids containing antibiotic resistance genes.

The stabilized CNPs on the nanofiber surface (Scaffold@CNPs) were tested as a potential biomedical application. Electrospun nanofibers have been widely used for skin tissue engineering and wound dressing due to their extracellular matrix mimicry, biodegradability, and biocompatibility [49]. In another study, nanofibers containing NPs such as silver, zinc oxide, and gold have been used for wound healing applications [49]. Then, the stabilization of NPs on the nanofiber surface helped the NPs last longer at the site of infection with their continuous release. Our study found that the CNPs were released during 9 days, and then the slow release of them assures a repeated administration might not be necessary. Also, in our study, the use of gelatin-PCL increased the scaffold degradation or biocompatibility of the nanocomposite, influencing the degradation behavior [49].

The SHV-betalactamase (*shv* gene [50, 51]), KPC-carbapenemase (*kpc* gene [52–54]), and metallo-β-lactamase IMP-1 (*imp* gene [55–57]) have been found in resistance strains of *P. aeruginosa* isolated from various hospitals. Therefore, the measure of the expression level of these genes allows to detect antibiotic resistance of *P. aeruginosa* [50–55, 57, 58].

Our qPCR results showed that the exposure of the strains to the Scaffold@CNPs affected the expression of all three genes, mainly by downregulating their expression. The highest reduction was observed on the expression of *shv* and *imp* compared to the expression of the *kpc*. Interestingly, although the soluble CNPs reduced the bacterial titer in the MIC test, it only downregulated the expression of *kpc* gene. The differences in the behaviour of both nanocomposites could result from the agglomeration of the soluble CNPs within 24 h. At the same time, the gradual release of the CNPs from the scaffold prevents them from agglomerating. The agglomeration of the soluble CNPs is supported by the fact that their zeta potential is + 18 mV, a value that suggests clumping. In a study reported by Abbas Fazal et al., the CNPs deposited on nano-sheets exhibited stronger antibacterial activity than the nanoparticles [59]. They showed a higher surface area, leading to a higher concentration of oxygen vacancies on the surface, which caused enhanced ROS generation. ROS has the key role in damage the bacterial membrane and is one of the main mechanisms of CNPs kill bacteria [60, 61].

On the other hand, simultaneous treatment of Scaffold@CNPs supplemented with antibiotics did not affect on the expression of any of the genes, and soluble CNPs along with antibiotics only reduced the expression of the *kpc* gene.

There are conflicting studies on the simultaneous effect of NPs and antibiotics. However, some studies reported that CNPs could act as antibiotic adjuvants to increase the effectiveness of antimicrobials and facilitate the entry of antibiotics into the cell by increasing the cell membrane permeability. But some studies are consistent with the results of our study and reported that the antibacterial effect of antibiotics could be dramatically reduced by concomitant treatment with CNPs, which may inhibit antibiotic uptake into the bacterial cell or disrupt antibiotic activity within the bacterial cell.

According to the survival results of HU2 fibroblast cells exposed to CNPs and Scaffold@CNPs, it seams the CNPs at low concentrations of nanoparticle, both soluble CNPs and stabilized on the scaffold, it has a cytotoxic effect on fibroblast cells, but with increasing concentration of nanoparticles, the toxicity decreases and at a concentration of 200 µg/ml reached the control group. The increase in cell growth with increasing concentration of CNPs is consistent with the results of study of Chigurupati et al. [62] that showed the growth rate of keratinocytes and fibroblasts cells which treated with 1 and 10 µM CNPs increased significantly compared to cultures treated with 500 nM or without CNPs. In this study, our goal was to find out that at concentrations that nanoparticles can kill *P.aeroginosa*, it has no toxic effect on healthy cells around the wound. In addition to this result, a decrease in the number of cells at lower concentrations was observed, which requires more observations and more detailed studies to confirm.

## Conclusions

The nanocomposites CNPs and Scaffold@CNPs showed potent anti-*Pseudomonas* activity. The. Scaffold@CNPs significantly downregulated the expression of three genes known as involved in the acquisition of antibacterial resistance. This property is significant as Scaffold@CNPs could be developed for topical applications or wound dressing. In addition, the slow release of the CNPs from the nanofiber represents a new modality for skin infection therapies.

## Data Availability

The data that support the findings of this study are available from the corresponding author (FR) on request.
